# Assessment of DNA Transfer on Drug Packages in Simulated Vehicular and Household Settings

**DOI:** 10.3390/genes16111264

**Published:** 2025-10-27

**Authors:** Xiaoyang Li, Yong Sheng Lee, Hui Wen Yeo, Marlene Abdul Mugni, Nurul Insyirah Binte Ishak, Sabrina Binte Mustaffa, Nadira Binte Murad, Nurulain Haziqah Binte Ngatimin, Christopher Kiu-Choong Syn

**Affiliations:** DNA Profiling Laboratory, Biology Division, Health Sciences Authority, 3 Biopolis Drive, Synapse, 03-14/15/16, Singapore 138623, Singapore

**Keywords:** DNA transfer, touch DNA, illicit drugs, drug packages, vehicles, households, activity-level evaluations

## Abstract

**Background**: DNA evidence can play a critical role during the investigation of illicit drugs cases. A key challenge, however, is in determining whether DNA profiles recovered on the evidence items, such as drug packages, arise from direct handling or indirect transfer. **Methods**: In this respect, we simulated common drug offense scenarios where illicit drugs were discovered inside vehicular or household settings that can be linked to the suspect. DNA transfer was evaluated based on two hypothetical propositions: (1) the individual’s DNA was directly deposited onto the mock drug packages through handling, or alternatively (2) DNA persisting on a particular surface had transferred onto the packages. For the direct transfer scenario, 15 participants were requested to pack the mock drug packages using their bare hands. For the indirect transfer scenario, DNA-free mock drug packages were placed at various locations in 15 vehicles and 15 bedrooms for different time intervals. Following each scenario, DNA samples were collected from multiple areas of the mock drug packages. DNA transfer was assessed based on quantification and profiling results, which were subsequently evaluated within a Bayesian network-based framework. **Results**: Higher DNA transfer frequencies onto the mock drug packages were observed in the direct-handling experiment compared to those from indirect transfer, suggesting that DNA transfer occurs to a higher degree from active contact. In direct-handling scenarios, the amount of DNA recovered from the handles of the carrier bags was much higher than that from the exterior bodies, indicating preferential DNA deposition at the contact areas. Conversely, the results from indirect transfer scenarios showed similar recovered DNA amounts between the handles and the exterior bodies of the carrier bags, with minimal transfer to the interior surfaces. These findings indicate that the likelihood of recovering DNA from specific areas of evidence items can be linked to the particular activities and transfer mechanisms involved. **Conclusions**: The findings of this study expand the empirical knowledge of DNA transfer processes across diverse forensic contexts.

## 1. Introduction

DNA evidence has long been an essential part of forensic investigations, aiding in the identification of individuals linked to criminal activities [1,2]. However, despite its widespread acceptance and utility in criminal investigations and court proceedings, the evaluation of DNA evidence at the activity level, especially in cases involving indirect DNA transfer, remains a complex challenge.

DNA can be deposited onto a surface via direct contact, indirect transfer, or even airborne dispersal [3,4,5]. There is extensive literature that explores the transfer, persistence, prevalence, and recovery of DNA [5,6,7]. For example, DNA can be transferred through routine interactions, such as handshakes or object sharing, and subsequently become deposited onto new surfaces [8,9]. Once deposited, it may persist for extended periods, creating opportunities for secondary DNA transfer, further complicating forensic interpretations [10,11]. In addition, numerous studies have reported detectable levels of background DNA on everyday objects and even in environmental dust, adding complexity to activity-level evaluations such as determining the timing and circumstances of DNA deposition [12,13,14,15]. Hence, the mere presence of an individual’s DNA on an evidence item cannot indicate ownership or direct involvement in any criminal activity. Rather, there is a need for careful evaluation of potential transfer mechanisms when assessing DNA evidence.

Recent publications have focused on the evaluation of DNA evidence in relation to activity-level propositions, with the aim of improving interpretations on how DNA persists and transfers in different contexts [16,17]. While a definitive distinction between direct and indirect transfer based on DNA evidence alone remains challenging, probabilistic tools such as Bayesian networks can provide structured frameworks to model complex DNA transfer scenarios [18,19]. These studies and analytical tools are important for assisting criminal investigators and courts in assessing the relevance and significance of DNA findings.

In the investigation of illicit drug offenses, a commonly encountered question is how DNA has been deposited onto the drug packages. Courts seek reliable references (e.g., empirical data) to determine whether DNA recovered on drug packages is a consequence of deliberate handling or indirect transfer. Recent studies have employed simulation experiments and Bayesian networks to assess activity-level propositions in such cases. Lee et al. demonstrated that both storage duration and shedder status could significantly influence DNA transfer onto mock drug packages within a personal bag [20]. Similarly, Fonneløp et al. assessed transfer probabilities in direct and indirect contact involving zip-lock drug bags [21]. In a more realistic household setting, Reither et al. examined both passive and active DNA transfer to the body and seal of zip-lock drug bags [22]. Additionally, various areas on evidence items have also been analyzed to establish links between individuals and different stages of drug handling [23,24]. These studies have collectively enhanced our understanding of DNA evidence in drug-related cases. However, additional empirical studies are needed to assess DNA transfer and persistence on drug-related items across diverse, case-relevant contexts to establish context-specific datasets to assist in forensic interpretations.

This study investigated DNA transfer in a commonly encountered scenario where illicit drugs were discovered at a location that could be linked to the suspect. While earlier studies usually examined DNA transfer within single environments, we evaluated indirect DNA transfer at six typical locations across two realistic environments, i.e., vehicle and household, frequently encountered in such offenses. A bedroom is more intimate, primarily accessed by the participant with occasional access by other family members. In contrast, the vehicle, while typically having a primary driver, could be accessed several times a week by different people (non-family members such as colleagues). As such, these two environments differ in accessibility, usage patterns, and, consequently, background DNA prevalence. Such a comparison can provide new insight into how environmental context influences DNA transfer and persistence on drug packages, reflecting situations frequently encountered in forensic casework. Additionally, we have included an assessment of direct DNA transfer during simulated drug-packing activities. The DNA transfer frequency dataset established through the study could support more informed and context-specific interpretation of DNA evidence. We also present a Bayesian network to provide a structured probabilistic framework for evaluating DNA evidence at the activity level.

## 2. Materials and Methods

### 2.1. Study Design

#### 2.1.1. Preparation of DNA-Free Mock Drug Packages for Vehicle and House Experiment

To prepare the DNA-free mock drug package, 200 g of salt was placed into a resealable plastic bag (approximately 16 cm × 9 cm), which was then placed inside a larger plastic carrier bag (approximately 52 cm × 31 cm).

The DNA-free mock drug packages were prepared by staff wearing full personal protective equipment (PPE), including shower caps, face masks, laboratory coats, sleeves, and gloves. Prior to preparation, five control samples were taken from each pack of resealable bags, and two control samples were taken from each pack of carrier bags at random to assess for background DNA. All control samples from resealable bags (*n* = 5) and carrier bags (*n* = 26) recorded 0 ng of DNA at real-time DNA quantitation, and no allelic peaks were detected.

#### 2.1.2. Vehicle

Fifteen participants, each with a vehicle used for at least 6 months prior to sampling, participated in this experiment. Four different locations within the vehicle were selected to store the DNA-free mock drug packages. The four locations were classified into two groups, open and enclosed spaces, based on the degree of accessibility of the locations. The open-space locations, which comprise (1) the rear passenger seat and (2) the floor beside the front passenger seat, are exposed and readily accessible within the vehicle. In contrast, the (3) glove compartment and (4) boot were deemed as enclosed-space locations as these areas required deliberate access.

One mock drug package was stored at each of the 4 different locations within each vehicle. The packages were stored for both a short (up to 3 h; specific durations detailed in Supplementary Table S1) and a long (2 days) duration. The two experiments with different storage durations were conducted separately for each vehicle, with an interval of at least 1 week. The handle of the carrier bag remained untied during the storage period, and participants were allowed to resume normal activities.

The detailed sample sizes for the vehicle experiment are listed in Table 1.

#### 2.1.3. House

Fifteen participants, each with a self-occupied bedroom where they had resided for at least 6 months prior to sampling, participated in this experiment. Similar to the vehicle study, one mock drug package was placed at an open space (e.g., on a table) while another mock drug package was placed in an enclosed space (e.g., inside a drawer) within the bedroom. The packages were stored at the selected locations for 2 days with the handle of the carrier bag untied, and participants were allowed to resume normal activities.

The detailed sample sizes for the house experiment are listed in Table 1.

#### 2.1.4. Non-DNA-Free Mock Drug Package

Fifteen participants washed their hands thoroughly before resuming normal activities for at least one hour before preparing the mock drug packages as described in Section 2.1.1 without wearing PPE. The participants further carried the carrier bag by holding the handle and walked for 5 min. These packages represent the scenario whereby a criminal, in the process of packing the illicit drugs into the plastic bags, directly transfers his/her DNA onto the drug package.

#### 2.1.5. Questionnaire

After each experiment, the participants completed a questionnaire to document potential factors affecting DNA transfer, such as the frequency of cleaning the studied surfaces, the frequency of usage by external individuals, and activities undertaken during the storage of mock drug packages.

Detailed information on the experimental conditions, including the specific storage locations of mock drug packages, the duration of storage, and participant responses from the post-experiment questionnaires is recorded in Supplementary Tables S1–S3.

### 2.2. Sample Collection

DNA samples were collected from the mock drug packages using a “double swabbing” method with sterile wet and dry cotton swabs.

For the DNA-free mock drug packages collected from the vehicles and houses, DNA samples were collected from the following areas: (1) the handle of the carrier bag, (2) the exterior body of the carrier bag, and (3) the interior of the carrier bag, together with the exterior of the resealable bag. Additionally, a background sample was collected from an area (approximately 10 cm × 10 cm) near each mock drug package at the end of the storage period.

For the non-DNA-free mock drug packages, DNA samples were collected from (1) the handle of the carrier bag, (2) the exterior body of the carrier bag, (3) the interior of the carrier bag, and (4) the exterior of the resealable bag.

### 2.3. DNA Analysis

DNA extraction was performed using the DNA IQ^TM^ Casework Extraction Kit and the DNA IQ^TM^ Casework Pro Kit on a Maxwell^®^ FSC instrument (Promega Corporation, Seoul, Republic of Korea). DNA yield was determined using the Quantifiler^®^ Trio DNA Quantification kit (Applied Biosystems, Warrington, UK; <1 pg/μL limit of detection, optimized to quantify DNA concentrations from 0.005 ng/uL to >50 ng/uL) on the QuantStudio^TM^ 7 Flex Real-Time PCR System (Applied Biosystems, Singapore). STR-PCR amplification was performed (29 cycles) with the GlobalFiler^TM^ PCR Amplification kit (Applied Biosystems, Warrington, UK) using 1 ng input DNA or a maximum of 15 µL DNA extract. Capillary electrophoresis was performed on a 3500xL Genetic Analyzer (Applied Biosystems, Ibaraki, Japan) with 3 µL of amplified product injected at 1.2 kV for 24 s. Results were analyzed using GeneMapper^®^ ID-X v1.7 software.

### 2.4. Definition of Person of Interest (POI)

Vehicle experiment: The POI for the DNA-free mock drug package was the participant who had been the primary driver of his/her vehicle for at least 6 months prior to this study.

House experiment: The POI for the DNA-free mock drug package was the participant who had been the primary occupant in his/her own bedroom for at least 6 months prior to this study.

Non-DNA-free mock drug package: The POI was the participant who prepared the package without wearing PPE.

### 2.5. DNA Profile Interpretation and Data Analysis

The Maximum Allele Count (MAC) method was used to determine the minimum Number of Contributors (NOC) for each profile as described [25]. Briefly, the initial number of contributors (n) was determined using the MAC method. When any locus showed a peak height ratio (PHR) below 10%, or more than 1 locus showed a PHR between 10 and 35%, the NOC was increased by 1 (from n to n + 1) to account for the observed peak height imbalance. In cases where all loci demonstrated PHRs exceeding 35%, the NOC (n) was maintained. Additionally, when the POI was determined as a contributor in a profile where at least 1 locus required an additional contributor to explain the result, the NOC was increased by one (from n to n + 1).

Likelihood ratios (LRs) and mixture proportions were calculated using EuroForMix version 3.2.0 to assess whether the POI could be included as a contributor to each DNA profile. The analysis was based on 2 competing hypotheses: the prosecution hypothesis (*H_p_*), which assumed the POI was a contributor, and the defense hypothesis (*H_d_*), which assumed the POI was not a contributor. To ensure a conservative approach, an inclusion threshold of “LR = 1000” was applied. The POI was determined as a contributor to a profile only when the LR exceeded 1000.

DNA profiles were deemed uninterpretable if fewer than 16 allelic peaks were detected or if the determined NOC exceeded 3. These profiles were further categorized into 3 groups: “no profile” (no allelic peak was detected), “inconclusive” (1–15 allelic peaks were detected), and “complex mixture” (NOC exceeded 3).

Interpretable DNA profiles were categorized into the groups of “POI-included” or “POI-excluded”, depending on whether the POI could be determined as a contributor. Mixture proportions calculated using EuroForMix were used to assess whether a major contributor could be assigned. A major contributor was defined as having a mixture proportion above 66.7% in a two-person mixture or above 50% in a three-person mixture, and the other contributors in the mixture profile were assigned as the minor contributor(s). Mixture profiles without a major contributor were considered to have equal contributions from all of the contributors. Accordingly, the “POI-included” group was further subdivided into “POI-Single” (POI as the sole contributor), “POI-Major” (POI as the major contributor), “POI-Equal” (POI as an equal contributor), and “POI-Minor” (POI as a minor contributor). The “POI-excluded” group was subdivided into “UP-Single” (unknown person as the sole contributor), “UP-Major” (a major unknown contributor was assigned), and “UP-Equal” (unknown persons as equal contributors), based on the NOC and/or whether a major contributor was determined.

DNA quantity data are presented as ranges, means, and medians throughout the results. In addition, variability measures of 95% confidence intervals (95% CI) are provided to illustrate the degree of variation among replicates where applicable.

A comprehensive dataset, including the DNA quantities and profile interpretation results for all of the samples, is provided in Supplementary Table S4.

Statistical analysis was performed using the non-parametric Mann–Whitney U test, with a significance threshold of *p* < 0.05.

## 3. Results

### 3.1. Background Samples

#### 3.1.1. Background Samples from the Vehicle Experiment

A total of 120 background samples were collected from the areas adjacent to the mock drug packages placed at the four locations (*n* = 15 per location per duration) within the vehicles, with DNA quantities ranging from 0 ng to 106 ng (mean of 3.20 ng; median of 0.66 ng; 95% CI: 1.30–5.11 ng). The experiment was conducted over both short-duration and long-duration storage periods to simulate typical short-distance transportation and extended storage scenarios, respectively. No significant difference was observed in the quantity of DNA for the samples collected from the same location between the two storage periods.

**Figure 1:**
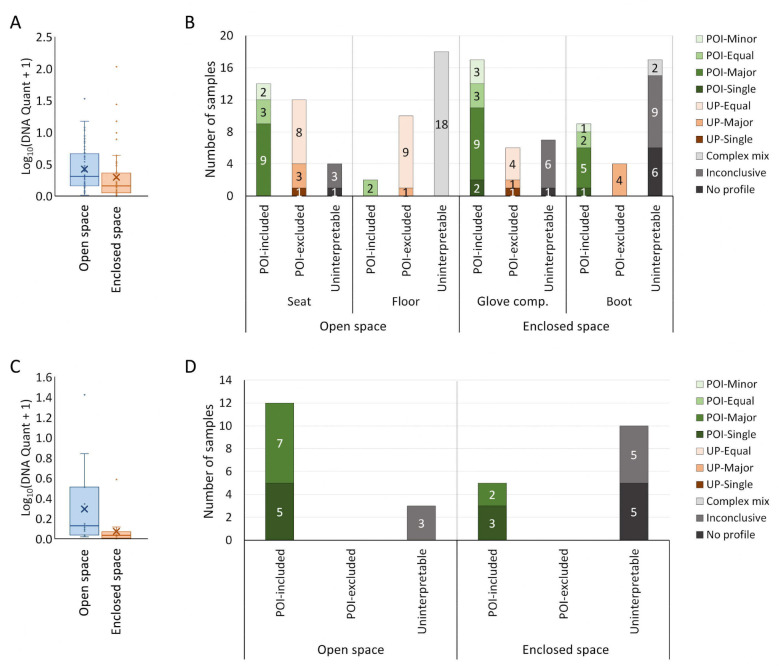
DNA recovery from the background samples in vehicle and house experiments (*n* = 120 for vehicle experiment; *n* = 30 for house experiment). Log-transformed DNA quantities recovered from the background samples in vehicle and house experiments are shown in (**A**) and (**C**), respectively. Box and whisker plots with interquartile ranges, where “×” represents the mean value. Compositions of DNA profiles from the background samples in vehicle and house experiments are shown in (**B**) and (**D**), respectively. The quantity and composition of DNA for each sample are listed in Supplementary Table S4.

As shown in Figure 1A, background samples from open-space locations yielded significantly higher amounts of DNA, ranging from 0.03 ng to 32.6 ng (mean of 2.90 ng; median of 1.02 ng; 95% CI: 1.63–4.18 ng). In contrast, DNA levels in background samples collected from enclosed-space locations were lower, with a range of 0 ng to 106 ng (mean of 3.50 ng; median of 0.42 ng; 95% CI: 0–7.15 ng). Within each group itself, there was no significant difference in the quantity of DNA recovered between the samples collected from the two different locations.

Open-space locations: From the 60 background samples collected from the open-space locations, 38 interpretable profiles were obtained (Figure 1B). The POI could be included as one of the contributors in 16 mixture profiles. The remaining 22 interpretable profiles were contributed by unknown individuals, including 1 single source profile and 21 mixtures.

Enclosed-space locations: Of the 60 background samples from the enclosed-space locations, 36 produced interpretable profiles (Figure 1B). The POI could be determined as the sole contributor for 3 profiles, while 23 profiles were DNA mixtures contributed by the POI and at least one unknown person. For the 10 profiles in which the POI was excluded as a contributor, 1 of them was a single source profile, while the remaining 9 were mixtures.

#### 3.1.2. Background Samples from the House Experiment

A total of 30 background samples were collected from open-space and enclosed-space locations within the houses, with DNA quantities ranging from 0 ng to 25.3 ng (mean of 1.43 ng; median of 0.14 ng; 95% CI: 0–3.17 ng).

Open-space locations: Background samples from the open-space locations (*n* = 15) yielded significantly higher amounts of DNA, ranging from 0.04 ng to 25.27 ng (mean of 2.60 ng; median of 0.34 ng; 95% CI: 0–6.17 ng; Figure 1C). Twelve of the 15 samples yielded interpretable profiles with the POI being the sole contributor in 5 profiles and as the major contributor in the remaining 7 (Figure 1D).

Enclosed-space locations: In comparison, the quantities of DNA recovered from the enclosed-space locations were significantly lower, ranging from 0 ng to 2.82 ng (mean of 0.27 ng; median of 0.08 ng; 95% CI: 0–0.66 ng; Figure 1C). Interpretable profiles could only be obtained from 5 of the 15 background samples from the enclosed-space locations, with the POI being the sole contributor in 3 profiles and as the major contributor in the remaining 2 (Figure 1D).

In comparison with the vehicle experiment, the profiles obtained from the background samples in the house experiment were generally simpler, with the POI being the sole or major contributor in all of the interpretable profiles.

### 3.2. Samples from the DNA-Free Mock Drug Packages

#### 3.2.1. Samples from the Vehicle Experiment

**Figure 2:**
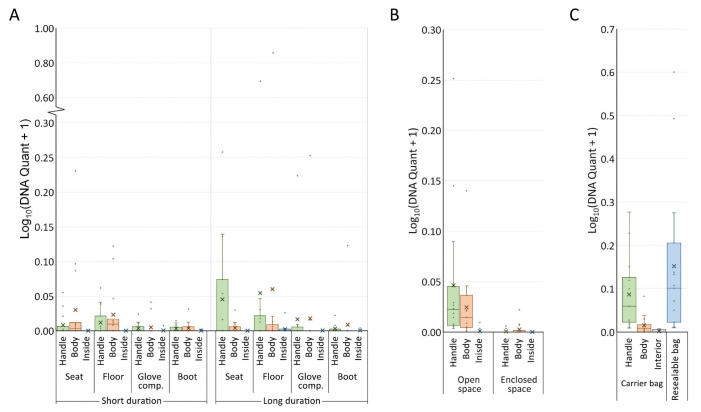
DNA recovery from the mock drug packages (*n* = 360 for the vehicle experiment; *n* = 90 for the house experiment; *n* = 60 for the direct-handling experiment). Log-transformed DNA quantities recovered from the mock drug packages in vehicle, house, and direct-handling experiments are shown in (**A**), (**B**), and (**C**), respectively. Box and whisker plots with interquartile ranges, where “×” represents the mean value. For (**A**,**B**), “Handle” = The handle of the carrier bag, “Body” = The exterior body of the carrier bag, and “Inside” = The interior of the carrier bag and the exterior of the resealable bag. For (**C**), “Resealable bag” = The exterior of the resealable bag. The quantity and composition of DNA for each sample are listed in Supplementary Table S4.

The study of DNA-free mock drug packages placed in four locations inside vehicles involved a total of 360 samples. DNA transfer was detected in 85 samples (Figure 2A, Supplementary Table S4). DNA transfer was considered to have occurred when a value above 0 was recorded during real-time DNA quantitation. The quantities of recovered DNA ranged from 0 ng to 6.17 ng (mean of 0.05 ng; median of 0 ng; 95% CI: 0.01–0.09 ng). DNA transfer was observed in 55 samples recovered from the open-space locations and 30 samples recovered from the enclosed-space locations.

To evaluate the effect of storage time on DNA transfer in real environments, this experiment was conducted twice for each vehicle with two different durations of storage time. Samples collected from identical areas on the drug packages placed at the same location showed no significant differences in DNA quantity between the two storage periods.

For mock drug packages recovered from the same location in the vehicle, no significant differences in DNA quantity were observed between samples recovered from the handle and from the exterior body of the carrier bag. For each mock drug package, the interior of the carrier bag and the exterior of the resealable bag were swabbed together to produce a single sample. Significantly lower amounts of DNA were recovered from these samples as compared to those recovered from the handle or exterior body of the carrier bag. Seven samples showed evidence of DNA transfer (in terms of quantifiable DNA), but only two samples produced DNA profiles with one and three allelic peaks, respectively.

Fifteen interpretable profiles were obtained from the samples recovered from the mock drug packages, with six from the short-duration experiment (Table 2) and nine from the long-duration experiment (Table 3). Most of the interpretable profiles (13 profiles) were recovered from open-space locations, while only two were from enclosed-space locations. Among the six interpretable profiles from the short-duration experiment, the POI could be included as the minor contributor in only one profile. The remaining five profiles, including three single source profiles and two mixtures, were attributed to unknown individuals. For the long-duration experiment, the POI was identified as the sole contributor in one profile, a minor contributor in another, and a major contributor in a third profile. The remaining six interpretable profiles originated from unknown contributors, comprising one single source profile and five mixtures.

#### 3.2.2. Samples from the House Experiment

In this experiment, a total of 90 samples were recovered from the mock drug packages, and DNA transfer was detected in 38 of them (Figure 2B, Supplementary Table S4). More DNA was recovered from the samples at open-space locations, with the recovered DNA quantities ranging from 0 ng to 0.78 ng (mean of 0.06 ng; median of 0.01 ng; 95% CI: 0.02–0.11 ng). DNA transfer was detected in only six samples from the enclosed-space locations, however (Figure 2B, Supplementary Table S4). The quantities of the recovered DNA ranged from 0 ng to 0.05 ng (mean of 0.002 ng; median of 0 ng; 95% CI: 0–0.01 ng). For the samples obtained from the same location, no significant differences were observed for the quantities of DNA recovered from the handle and those from the exterior body of the carrier bag. Similar to the vehicle experiment, very few DNA transfer events were detected in the samples (three samples) collected from the interior of the carrier bag and the exterior of the resealable bag, and no allelic peak was obtained in these profiles.

Sixteen interpretable profiles were generated from the mock drug packages stored at open-space locations, with the POI determined as the sole contributor for nine of them and the major contributor for the remaining seven (Table 4). In contrast, only four inconclusive DNA profiles were generated from the enclosed-space locations.

### 3.3. Samples from the Non-DNA-Free Mock Drug Packages

This evaluation of direct DNA transfer by the participant who had packed the mock drug package without wearing PPE involved a total of 60 samples. DNA transfer was detected in 47 samples (Figure 2C, Supplementary Table S4). The quantities of DNA recovered from the handle of the carrier bag ranged from 0.02 ng to 0.89 ng (mean of 0.24 ng; median of 0.15 ng; 95% CI: 0.10–0.39 ng). It was significantly higher than that from the exterior body of the carrier bag, which ranged from 0 ng to 0.21 ng (mean of 0.04 ng; median of 0.02 ng; 95% CI: 0.01–0.07 ng). Two samples were separately collected from the exterior of the resealable bag and the interior of the carrier bag. DNA quantities recovered from the exterior of the resealable bag ranged from 0.02 ng to 2.98 ng (mean of 0.56 ng; median of 0.26 ng; 95% CI: 0.09–1.03 ng). DNA transfer to the interior of the carrier bag was minimal, with only six samples yielding detectable DNA amounts and only one producing an inconclusive profile.

For the exterior side of the carrier bag, 10 interpretable profiles were obtained from the handle, and 2 interpretable profiles were obtained from the body (Table 5). Nine of these profiles were solely contributed by the POI, while the POI was determined as the major contributor in the remaining three. Meanwhile, ten interpretable profiles were generated from the exterior of the resealable bag, with the POI determined as the sole contributor in eight profiles and the major contributor in the other two.

### 3.4. Evaluation of Case Scenarios

**Figure 3:**
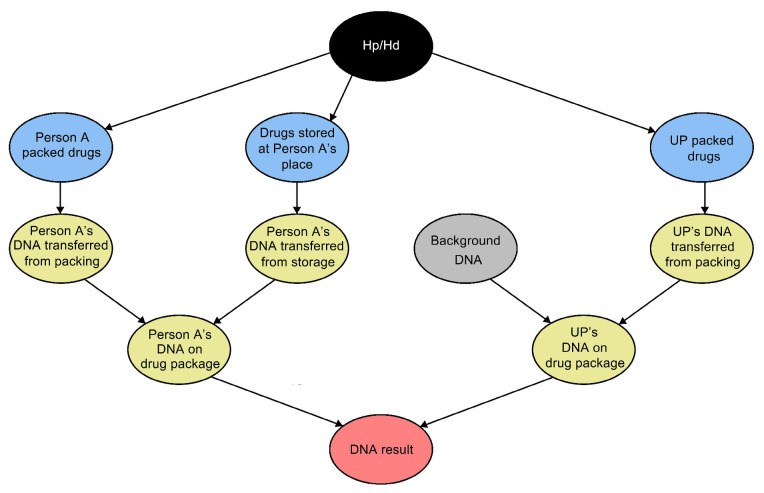
Bayesian network constructed for the case scenarios. Nodes are coloured so that black represents the main propositional node, blue represents the activity node, grey represents the root node, yellow represents the intermediate results node, and pink represents the findings node. “UP” = Unknown person.

The collected dataset could be used for the evaluation of DNA evidence from illicit drug crimes. To illustrate this application, 2 examples are presented below, whereby evidence is recovered from a household (Case Scenario 1) or a vehicular (Case Scenario 2) setting. In this respect, a Bayesian network is first constructed in Figure 3 as previously reported [26].

Case Scenario 1: A carrier bag was discovered inside the drawer of Person A’s bedroom, and a resealable bag containing illicit drugs was found inside the carrier bag. Upon questioning, Person A claimed that he was not aware of the bag and had never seen it before. A single source profile was recovered from the exterior body of the carrier bag, and Person A could not be excluded as the contributor for the profile.

The activity level propositions are as follows:

(1) *H_p_*: Person A packed the drugs and kept the drug package in his bedroom
(2) *H_d_*: An unknown person packed the drugs and kept the drug package in Person A’s bedroom

We followed the calculation approaches as described [21]. The variables are listed in Table 6.

To illustrate a simple evaluation of the DNA evidence, only the presence or absence of DNA from the POI and unknown person(s) is considered here. The equations used are as follows:

(1) When the POI is observed as the contributor in a single source profile:
  $$LR=\frac{\left(s\times \left(1-t\right)+t\right)\times (1-b)}{s\times \left(1-t^{\prime }\right)\times \left(1-b\right)}=\frac{s\times \left(1-t\right)+t}{s\times \left(1-t^{\prime }\right)}$$
(2) When the POI is observed as a contributor in a mixture profile:
  $$LR=\frac{\left(s\times \left(1-t\right)+t\right)\times b}{s\times (t^{\prime }+b\times \left(1-t^{\prime }\right))}$$

The probabilities of direct and indirect DNA transfer are calculated based on the dataset collected from each specific scenario in this study, while the probability of background DNA transfer is calculated based on the detection of non-POI contributor(s) in the samples from all three experiments. To account for the limited sample size, a prior count of “1” is added to the observed counts when calculating the probability of each observation.

For Case Scenario 1, *t* = *t′* = (2 + 1)/(15 + 2) ≈ 0.18, *s* = (0 + 1)/(15 + 2) ≈ 0.06.

$${\mathrm{LR}}_{1}=\frac{0.06\times \left(1-0.18\right)+0.18}{0.06\times (1-0.18)}\approx 4.66$$

Case Scenario 2: A carrier bag was discovered on the rear seat of a vehicle driven by Person A, and a resealable bag containing illicit drugs was found inside the carrier bag. Upon questioning, Person A claimed that he was not aware of the bag and had never seen it before. Person A also claimed that the vehicle was a rideshare vehicle and the bag was likely left there by the last passenger a few hours ago. A mixture profile was recovered from the handle of the carrier bag, and Person A could not be excluded as a contributor to the profile.

The activity level propositions are as follows:

(1) *H_p_*: Person A packed the drugs and kept the drug package in his vehicle
(2) *H_d_*: An unknown person packed the drugs and kept the drug package in Person A’s vehicle

For Case Scenario 2, *b* = (13 + 1)/(165 + 2) ≈ 0.08, *t* = *t′* = (10 + 1)/(15 + 2) ≈ 0.65, *s* = (0 + 1)/(15 + 2) ≈ 0.06.

$${\mathrm{LR}}_{2}=\frac{\left(0.06\times \left(1-0.65\right)+0.65\right)\times 0.08}{0.06\times (0.65+0.08\times \left(1-0.65\right))}\approx 1.32$$

Based on the European Network of Forensic Science Institutes guideline for evaluative reporting [27], LR_1_ provides weak support for *H_p_* rather than *H_d_*, while LR_2_ suggests that the DNA result does not support one proposition over the other.

It should be noted that the preceding example only evaluated the DNA evidence from 1 sample recovered from 1 area of the drug package. In the event that DNA evidence is recovered from multiple areas (e.g., the handle of the carrier bag, the body of the carrier bag, and the exterior of the resealable bag), our dataset from these different areas of the evidence can be combined to calculate LRs given activity-level propositions and to draw more robust conclusions based on the case context.

## 4. Discussion

### 4.1. DNA Transfer and Persistence in Different Environments

This study simulated common forensic case scenarios involving illicit drug packages found in vehicles and households. Previous research has shown that DNA collected from an area adjacent to where the evidence item makes direct contact could serve as a control, which can indicate the background DNA present on the actual area in direct contact with the evidence item [28,29,30]. Control samples collected from these adjacent areas may help to distinguish which DNA components on the evidence item likely stem from the activity of interest versus unrelated background, thereby assisting in activity-level interpretation. In our study, background samples were collected from four locations in vehicles and two locations in households. Background DNA profiles recovered from vehicles exhibited greater complexity and diversity compared to those from households, likely due to the higher frequency of different individuals entering and using the vehicle over time, whereas household settings offer more privacy and limited access. Consistently, DNA profiles recovered from mock drug packages stored in the households were also less complex than those from the vehicles, with the POI determined as either the sole or major contributor in all of the interpretable profiles.

Significantly higher amounts of background DNA were recovered from the open-space locations than from the enclosed-space locations in both vehicle and household experiments. This is likely due to the increased exposure and accessibility of open spaces, allowing for the accumulation of shed DNA from individuals using the area. In contrast, enclosed compartments would reduce primary DNA deposition, although not completely preventing DNA transfer. These findings align with previous studies indicating that DNA accumulates more readily on frequently handled or exposed surfaces [31]. In addition, more DNA transfer events were also observed at open-space locations, and higher DNA quantities were recovered from mock drug packages stored at these locations in both vehicle and household experiments. These results suggest that exposure and accessibility of space can have a significant impact on DNA accumulation and subsequent secondary transfer.

A notable portion of samples from the mock drug packages contained DNA of unknown individuals. Over 93% of the reportable profiles in the vehicle experiment and over 43% of the reportable profiles in the household experiment were contributed by one or more unknown individuals. The presence of DNA profiles from unknown contributors raises important considerations regarding background DNA in drug-related cases. One likely explanation is the indirect transfer of background DNA, whereby shed skin cells or other biological materials previously deposited onto surfaces in these environments are subsequently transferred to the evidence [30,32,33,34]. For example, it could be expected that DNA from other drivers, passengers, or maintenance service personnel would be present in high-touch point areas in the vehicle, which would, later, be indirectly transferred onto the drug packages. This may be particularly relevant in forensic investigations where suspects deny handling evidence though their DNA profiles could be recovered.

Although the carrier bags and resealable bags used in this study were confirmed to be DNA-free, real-world materials may have been handled by multiple individuals during manufacturing, packing, or retail distribution. If DNA is deposited during these processes, it can persist on the surface and be detected during forensic analysis. These potentially pre-existing pieces of DNA can make forensic DNA analysis more challenging and highlight the need for the evaluation of DNA evidence under specific case contexts.

In the vehicle experiment, both a short-duration and a long-duration storage period were used to simulate typical transportation and extended storage scenarios, respectively. Notably, no significant differences in recovered DNA quantities from the mock drug packages were observed between the two storage periods. This suggests that passive storage conditions alone did not substantially increase DNA accumulation over the 2-day timeframe of our study. Instead, the amount of DNA present on a recovered item may be more indicative of the mode of transfer (e.g., direct handling vs. indirect transfer) rather than the duration of contact. Temperature, humidity, and airflow were not formally recorded or controlled in this study, but all of the experiments were conducted under stable indoor or vehicular conditions. Further research is necessary to evaluate whether varying environmental factors (e.g., temperature and humidity) would influence DNA persistence and transfer. The maximum storage duration of two days in this study limit conclusions that may be drawn regarding long-term DNA persistence, as environmental fluctuations and extended exposure may further influence DNA degradation and subsequent forensic detection. Future studies incorporating longer storage periods and varied environmental conditions would improve ecological validity and provide a more comprehensive understanding of DNA persistence in other case scenarios.

### 4.2. DNA Transfer to Different Areas of the Mock Drug Packages

To reflect real-world drug packing and trafficking methods, a carrier bag was used as the secondary packaging of the mock drug package in this study. This simulated a common concealment practice and allowed for the study of DNA transfer across multiple packaging layers.

In the vehicle and household experiments, participants did not have direct contact with the resealable bag placed inside the carrier bag. This simulated a scenario where the suspect claimed that he had not come into contact with the illicit drugs within the packaging. In this respect, we examined whether indirect DNA transfer could be detected on the resealable bag inside the carrier bag without there being any direct handling. Despite the handle of the carrier bag being left untied throughout the study, there were very few DNA transfer events detected on the interior of the carrier bags and the exterior of the resealable bags (9/150 samples; 6%), and no reportable profiles were obtained.

### 4.3. Comparison Between Direct Handling and Indirect Transfer

#### 4.3.1. Rate of DNA Transfer Events

The highest rate of DNA transfer events was observed from the non-DNA-free mock drug packages (representing the scenario whereby the POI packed and carried the drugs), with interpretable DNA profiles obtained from 10 of the 15 samples (67%) recovered from both the exterior of the resealable bags and the handles of the carrier bags. In comparison, no interpretable DNA profiles were obtained from the exterior of the resealable bags in both the vehicle and house experiments, while interpretable DNA profiles were only obtained from 7% and 23% of the samples recovered from the handles of the carrier bags in the vehicle and house experiments, respectively. This suggests that direct handling is still the primary process leading to DNA transfer.

#### 4.3.2. Complexity of DNA Profiles

In the direct-handling scenario, the POI was determined as the sole or major contributor to all interpretable DNA profiles. This contrasted with the drug packages passively stored in vehicles and households, where more complex DNA profiles were recovered, which is likely a reflection of the background DNA composition of the corresponding environments.

#### 4.3.3. Quantity of DNA Recovered

In both vehicle and household environments, no significant differences were observed between the quantities of DNA recovered from the handles and the exterior bodies of the carrier bags, indicating that DNA transfer was influenced by the area of contact rather than any inherent preference for specific bag locations under these passive contact scenarios. In contrast, the amounts of DNA recovered from the handles were approximately 6.6 times higher than that from the exterior bodies of the carrier bags in the direct-handling scenario, suggesting deliberate handling could lead to concentrated DNA deposition in specific areas such as the handles. This aligns with previous studies showing that DNA samples recovered from different areas of a drug package can yield varying evidential values and may be linked to distinct stages in drug packing and trafficking [23,24]. In practical terms, our findings suggest that forensic practitioners should prioritize sampling from high-contact areas (e.g., the interior of the packaging and bag handles) separately to potentially identify individuals who may be involved in different stages of the activities (e.g., packing vs. trafficking of drugs).

These results indicate that the DNA recovered from handled items is more likely to be attributable to the direct handler; however, the possibility of indirect DNA transfer via shared surfaces or other means should not be overlooked. Forensic practitioners would need to assess the transfer, persistence, and recovery of DNA evidence before drawing conclusions.

### 4.4. Interpretation Challenges and Future Research

In this study, a conservative threshold of “LR = 1000” was used to determine whether the POI could be included as a contributor for the obtained profiles. The selection of an inclusion threshold is a laboratory- or case-specific decision that can be influenced by legal, scientific, and practical considerations. Laboratories must account for factors such as sample quality and mixture complexity to balance inclusion and exclusion risks while minimizing interpretation errors. Therefore, forensic practitioners are encouraged to choose the LR threshold based on laboratory validation studies rather than arbitrary numerical limits. Additionally, although mixture proportions generated using probabilistic genotyping software such as EuroForMix offer valuable insight into the individual contributions in a DNA mixture, they must be used cautiously. Various factors, such as stochastic effects, allele sharing, and DNA degradation, can affect estimation accuracy. Practitioners are advised to treat such mixture proportions as supportive indicators rather than quantitative measures during activity-level evaluations.

Beyond determining the source of DNA, courts increasingly require activity-level evaluations to assess how DNA was deposited and whether its presence aligns with alleged criminal activities. This is crucial for ensuring accurate interpretations and preventing wrongful convictions. Despite its importance, activity-level evaluation presents significant challenges, especially when complex mixtures and indirect DNA transfer are involved. To address these challenges and support the probabilistic assessment of competing propositions (e.g., is the POI directly involved in the alleged activity or not?), simulation studies that replicate real-world crime scenarios are essential. These studies enable forensic practitioners to evaluate different DNA transfer mechanisms systematically and refine the probabilistic models used in forensic casework. Despite their usefulness, the controlled nature of activity-level studies cannot fully capture the infinite spectrum of factors such as temperature, humidity, airflow, and surface material that can influence DNA transfer, prevalence, persistence, and recovery [3,4,5,6,7]. Future developments in advanced probabilistic models and artificial intelligence tools may be needed to integrate various DNA transfer scenarios and enhance forensic interpretations.

It should be noted that the experiments were conducted within a single laboratory setting. Although all procedures followed validated forensic protocols, inter-laboratory differences in handling practices and environmental conditions may influence DNA transfer outcomes. In this respect, future collaborative or inter-laboratory studies would be particularly valuable to ascertain the applicability of these findings in more varied forensic contexts.

It should be noted that the present study involved a cohort of 15 volunteers recruited from the same institution, resulting in a relatively homogeneous cohort. Such a design with limited biological (e.g., shedder status and skin condition), behavioral, and/or environmental (e.g., temperature, humidity, and airflow) variables that could influence DNA transfer would maximize the consistency of experimental procedures but may reduce the generalization of the findings. Future studies with larger and more demographically diverse participants could help capture a wider range of inter-individual variability, though the experimental design would concomitantly increase in complexity.

## 5. Conclusions

The present study simulated direct handling and indirect DNA transfer scenarios in real-world vehicular and household settings frequently encountered in illicit drug offenses. We compared DNA transfer between direct handling and passive storage at both open-space and enclosed-space locations. The results demonstrated that direct handling resulted in higher DNA transfer frequencies than indirect transfer, with DNA concentrated at contact areas such as the handles of carrier bags. In contrast, results from the indirect transfer experiment showed similar recovered DNA levels across different exterior areas of carrier bags with minimal transfer to interior surfaces, indicating that DNA deposition patterns could reflect specific transfer mechanisms. These results on DNA persistence and transfer would assist activity-level evaluation of forensic DNA evidence, expanding empirical data across different case contexts and emphasizing the importance of considering transfer mechanisms in DNA result interpretation. The generated dataset can be further integrated into probabilistic evaluation frameworks or LR databases to promote evidence-based evaluation of DNA transfer in casework.

## Figures and Tables

**Table 1:** Sample sizes for vehicle and house experiments. “Seat” = Rear passenger seat; “Floor” = Floor beside the front passenger seat; “Glove” = Glove compartment.

Experiment	Duration	Subgroup	Location	Sample Size (*n*)	Sampled Areas
Vehicle	Up to 3 h	Open space	Seat	15	(1) Background area (*n* = 15); (2) The handle of the carrier bag (*n* = 15); (3) The exterior body of the carrier bag (*n* = 15); (4) The interior of the carrier bag together with the exterior of the resealable bag (*n* = 15)
			Floor	15	
		Enclosed space	Glove	15	
			Boot	15	
	2 days	Open space	Seat	15	
			Floor	15	
		Enclosed space	Glove	15	
			Boot	15	
House	2 days	Open space	-	15	
		Enclosed space	-	15	

**Table 2:** Interpretation of DNA profiles for samples recovered from the mock drug packages in the short-duration vehicle experiment. “Handle” = The handle of the carrier bag; “Body” = The exterior body of the carrier bag; “Inside” = The interior of the carrier bag and the exterior of the resealable bag.

		Seat			Floor			Boot			Glove Compartment		
		Handle	Body	Inside	Handle	Body	Inside	Handle	Body	Inside	Handle	Body	Inside
POI-included	POI-Single												
	POI-Major												
	POI-Equal												
	POI-Minor		1										
POI-excluded	UP-Single	1			1	1							
	UP-Major		1										
	UP-Equal					1							
Uninterpretable	No profile	13	12	15	11	11	15	13	13	15	13	14	15
	Inconclusive	1	1		3	2		2	2		2	1	
	Complex mixture												

**Table 3:** Interpretation of DNA profiles for samples recovered from the mock drug packages in the long-duration vehicle experiment. “Handle” = The handle of the carrier bag; “Body” = The exterior body of the carrier bag; “Inside” = The interior of the carrier bag and the exterior of the resealable bag.

		Seat			Floor			Boot			Glove Compartment		
		Handle	Body	Inside	Handle	Body	Inside	Handle	Body	Inside	Handle	Body	Inside
POI-included	POI-Single	1											
	POI-Major	1											
	POI-Equal												
	POI-Minor					1							
POI-excluded	UP-Single	1											
	UP-Major	2			1				1				
	UP-Equal											1	
Uninterpretable	No profile	8	12	15	8	11	13	15	14	15	13	14	15
	Inconclusive	2	3		6	3	2				2		
	Complex mixture												

**Table 4:** Interpretation of DNA profiles for samples recovered from the mock drug packages in the house experiment. “Handle” = The handle of the carrier bag; “Body” = The exterior body of the carrier bag; “Inside” = The interior of the carrier bag and the exterior of the resealable bag.

		Open Space			Enclosed Space		
		Handle	Body	Inside	Handle	Body	Inside
POI-included	POI-Single	4	5				
	POI-Major	3	4				
	POI-Equal						
	POI-Minor						
POI-excluded	UP-Single						
	UP-Major						
	UP-Equal						
Uninterpretable	No profile	2	1	15	12	14	15
	Inconclusive	6	5		3	1	
	Complex mixture						

**Table 5:** Interpretation of DNA profiles for samples recovered from the mock drug packages in the direct-handling experiment.

		Carrier Bag			Resealable Bag
		Handle	Body	Interior	Exterior
POI-included	POI-Single	7	2		8
	POI-Major	3			2
	POI-Equal				
	POI-Minor				
POI-excluded	UP-Single				
	UP-Major				
	UP-Equal				
Uninterpretable	No profile		6	14	1
	Inconclusive	5	7	1	4
	Complex mixture				

**Table 6:** Definitions of the variables used in the LR calculation.

Variable	Definition
*t*	Probability of direct transfer, persistence, and recovery of DNA from the POI (under *H_p_* only).
*t’*	Probability of direct transfer, persistence, and recovery from an unknown person (under *H_d_* only).
*s*	Probability of indirect transfer, persistence, and recovery (under *H_p_* and *H_d_*).
*b*	Probability of recovering background (foreign) DNA (under *H_p_* and *H_d_*).

## Data Availability

The original contributions presented in this study are included in the article/Supplementary Materials. Further inquiries can be directed to the corresponding author.

## Supplementary Materials

The following supporting information can be downloaded at https://www.mdpi.com/article/10.3390/genes16111264/s1. Table S1: Questionnaire data related to the vehicle experiment; Table S2: Questionnaire data related to the house experiment; Table S3: Questionnaire data related to the direct-handling experiment; Table S4: Raw data of DNA quantification and profile.

## Abbreviations

The following abbreviations are used in this manuscript:

PPE	Personal protective equipment
POI	Person of interest
MAC	Maximum Allele Count
NOC	Number of Contributors
PHR	Peak height ratio

## References

[1] van Oorschot R.A.H., Ballantyne K.N., Mitchell R.J. (2010). Forensic trace DNA: A review. Investig. Genet..

[2] Meakin G., Jamieson A. (2013). DNA transfer: Review and implications for casework. Forensic Sci. Int. Genet..

[3] van Oorschot R.A.H., Szkuta B., Meakin G.E., Kokshoorn B., Goray M. (2019). DNA transfer in forensic science: A review. Forensic Sci. Int. Genet..

[4] Gosch A., Courts C. (2019). On DNA transfer: The lack and difficulty of systematic research and how to do it better. Forensic Sci. Int. Genet..

[5] van Oorschot R.A.H., Meakin G.E., Kokshoorn B., Goray M., Szkuta B. (2021). DNA Transfer in Forensic Science: Recent Progress towards Meeting Challenges. Genes.

[6] Burrill J., Daniel B., Frascione N. (2019). A review of trace “Touch DNA” deposits: Variability factors and an exploration of cellular composition. Forensic Sci. Int. Genet..

[7] Sessa F., Pomara C., Esposito M., Grassi P., Cocimano G., Salerno M. (2023). Indirect DNA Transfer and Forensic Implications: A Literature Review. Genes.

[8] Lowe A., Murray C., Whitaker J., Tully G., Gill P. (2002). The propensity of individuals to deposit DNA and secondary transfer of low level DNA from individuals to inert surfaces. Forensic Sci. Int..

[9] Goray M., van Oorschot R.A. (2015). The complexities of DNA transfer during a social setting. Leg. Med..

[10] Raymond J.J., van Oorschot R.A., Gunn P.R., Walsh S.J., Roux C. (2009). Trace evidence characteristics of DNA: A preliminary investigation of the persistence of DNA at crime scenes. Forensic Sci. Int. Genet..

[11] Kaesler T., Kirkbride K.P., Linacre A. (2023). Persistence of touch DNA on commonly encountered substrates in different storage conditions. Forensic Sci. Int..

[12] Port N.J., Bowyer V.L., Graham E.A., Batuwangala M.S., Rutty G.N. (2006). How long does it take a static speaking individual to contaminate the immediate environment?. Forensic Sci. Med. Pathol..

[13] Toothman M.H., Kester K.M., Champagne J., Cruz T.D., Street W.S., Brown B.L. (2008). Characterization of human DNA in environmental samples. Forensic Sci. Int..

[14] van den Berge M., Ozcanhan G., Zijlstra S., Lindenbergh A., Sijen T. (2016). Prevalence of human cell material: DNA and RNA profiling of public and private objects and after activity scenarios. Forensic Sci. Int. Genet..

[15] Atkinson K., Arsenault H., Taylor C., Volgin L., Millman J. (2022). Transfer and persistence of DNA on items routinely encountered in forensic casework following habitual and short-duration one-time use. Forensic Sci. Int. Genet..

[16] Biedermann A., Champod C., Jackson G., Gill P., Taylor D., Butler J., Morling N., Hicks T., Vuille J., Taroni F. (2016). Evaluation of Forensic DNA Traces When Propositions of Interest Relate to Activities: Analysis and Discussion of Recurrent Concerns. Front. Genet..

[17] Taylor D., Kokshoorn B., Biedermann A. (2018). Evaluation of forensic genetics findings given activity level propositions: A review. Forensic Sci. Int. Genet..

[18] Biedermann A., Taroni F. (2012). Bayesian networks for evaluating forensic DNA profiling evidence: A review and guide to literature. Forensic Sci. Int. Genet..

[19] Schaapveld T.E.M., Opperman S.L., Harbison S. (2019). Bayesian networks for the interpretation of biological evidence. WIREs Forensic Sci..

[20] Lee L.Y.C., Lee Y.S., Tan J., Lee J.Y., Syn C.K. (2024). A study of DNA transfers onto plastic packets placed in personal bags. J. Forensic Sci..

[21] Fonneløp A.E., Faria S., Shanthan G., Gill P. (2021). Who Packed the Drugs? Application of Bayesian Networks to Address Questions of DNA Transfer, Persistence, and Recovery from Plastic Bags and Tape. Genes.

[22] Reither J.B., van Oorschot R.A.H., Durdle A., Szkuta B. (2023). DNA transfer to placed, stored, and handled drug packaging and knives in houses. Forensic Sci. Int. Genet..

[23] Stefanović A., Šorgić D., Cvetković N., Antović A., Ilić G. (2024). Precision touch DNA sampling on plastic bag knots for improved profiling of packer and holder contributions. Forensic Sci. Int. Genet..

[24] Nolan M., Linacre A. (2025). Illicit drug distribution: Evaluation of DNA transfer between ziplock bags and capsules. Forensic Sci. Int. Genet..

[25] Szkuta B., Ballantyne K.N., van Oorschot R.A.H. (2017). Transfer and persistence of DNA on the hands and the influence of activities performed. Forensic Sci. Int. Genet..

[26] Taylor D., Biedermann A., Hicks T., Champod C. (2018). A template for constructing Bayesian networks in forensic biology cases when considering activity level propositions. Forensic Sci. Int. Genet..

[27] European Network of Forensic Science Institutes (2015). ENFSI Guideline for Evaluative Reporting in Forensic Science—Strengthening the Evaluation of Results Across Europe (STEOFRAE). https://enfsi.eu/wp-content/uploads/2016/09/m1_guideline.pdf.

[28] European Network of Forensic Science Institutes (2022). Best Practice Manual for Scene of Crime Examination. https://enfsi.eu/wp-content/uploads/2021/12/BPM-SOC-01-v.2021115_final.pdf.

[29] van den Berge M., van de Merwe L., Sijen T. (2017). DNA transfer and cell type inference to assist activity level reporting: Post-activity background samples as a control in dragging scenario. Forensic Sci. Int. Genet. Suppl. Ser..

[30] Reither J.B., Gray E., Durdle A., Conlan X.A. (2021). Investigation into the prevalence of background DNA on flooring within houses and its transfer to a contacting surface. Forensic Sci. Int..

[31] Zacher M., van Oorschot R.A.H., Handt O., Goray M. (2024). Transfer and persistence of intruder DNA within an office after reuse by owner. Forensic Sci. Int. Genet..

[32] Szkuta B., Reither J.B., Conlan X.A., van Oorschot R.A.H. (2019). The presence of background DNA on common entry points to homes. Forensic Sci. Int. Genet. Suppl. Ser..

[33] Boyko T., Mitchell R.J., van Oorschot R.A.H. (2019). Prevalence of DNA from the driver, passengers and others within a car of an exclusive driver. Aust. J. Forensic Sci..

[34] De Wolff T.R., Aarts L.H.J., van den Berge M., Boyko T., van Oorschot R.A.H., Zuidberg M., Kokshoorn B. (2021). Prevalence of DNA of regular occupants in vehicles. Forensic Sci. Int..

